# Microbial Communities of Three Sympatric Australian Stingless Bee Species

**DOI:** 10.1371/journal.pone.0105718

**Published:** 2014-08-22

**Authors:** Sara D. Leonhardt, Martin Kaltenpoth

**Affiliations:** 1 Department of Animal Ecology and Tropical Biology, University of Würzburg, Würzburg, Germany; 2 Insect Symbiosis Research Group, Max Planck Institute for Chemical Ecology, Jena, Germany; Universidade de São Paulo, Faculdade de Filosofia Ciências e Letras de Ribeirão Preto, Brazil

## Abstract

Bacterial symbionts of insects have received increasing attention due to their prominent role in nutrient acquisition and defense. In social bees, symbiotic bacteria can maintain colony homeostasis and fitness, and the loss or alteration of the bacterial community may be associated with the ongoing bee decline observed worldwide. However, analyses of microbiota associated with bees have been largely confined to the social honeybees (*Apis mellifera*) and bumblebees (*Bombus* spec.), revealing – among other taxa – host-specific lactic acid bacteria (LAB, genus *Lactobacillus*) that are not found in solitary bees. Here, we characterized the microbiota of three Australian stingless bee species (Apidae: Meliponini) of two phylogenetically distant genera (*Tetragonula* and *Austroplebeia)*. Besides common plant bacteria, we find LAB in all three species, showing that LAB are shared by honeybees, bumblebees and stingless bees across geographical regions. However, while LAB of the honeybee-associated Firm4–5 clusters were present in *Tetragonula*, they were lacking in *Austroplebeia*. Instead, we found a novel clade of likely host-specific LAB in all three Australian stingless bee species which forms a sister clade to a large cluster of Halictidae-associated lactobacilli. Our findings indicate both a phylogenetic and geographical signal of host-specific LAB in stingless bees and highlight stingless bees as an interesting group to investigate the evolutionary history of the bee-LAB association.

## Introduction

Mutualistic interactions are widespread in the animal and plant kingdoms and have left their footprints in the evolutionary history of many organisms. One of the most common groups of mutualists associated with multicellular organisms are bacteria, which are particularly prevalent across insects [1]. Mutualistic bacteria can provide a range of ecological benefits to their insect hosts, including nutritional upgrading of deficient diets, degradation of dietary polymers, and defense against antagonists (reviewed by [2]).

Bees represent an ecologically and economically important group of insects due to their functional role as pollinators in most ecosystems. Lately, they have declined in both abundance and species richness [3]–[6], with negative consequences for the quality and stability of pollination services to wild plants and agricultural crops [7]–[12]. Among bees, the (highly) social species play a particularly important role as pollinators due to the sheer numbers of foragers from single colonies, the diversity of species in some ecosystems (particularly in the tropics: [13]), the individual flower constancy (e.g., in bumblebees: [14],[15],[16]), the early onset of foraging (e.g., bumblebees: [17]) and year-long foraging in many species.

In this context, the microbial community associated with social bees has received considerable attention, and previous studies found a consistent core microbiota across honeybees and bumblebees [18]–[20]. While some of these symbiotic microbes have been hypothesized to aid in nutrient acquisition [21], others play an important role for the social immunity of bee colonies [21]–[23]. This function may be particularly relevant, as the diversity of immune-related genes is strongly reduced in honeybees (and likely other eusocial bee species) compared to other insects [24]. In the bumblebee *Bombus terrestris*, gamma- and betaproteobacterial symbionts convey resistance against an intestinal parasitic protozoan (*Crithidia bombi*) that negatively impacts the bees' fecundity [25]. Within honeybee nests, several *Bacillus* strains, actinomycetes, as well as some fungal associates have been isolated from pollen, honey and nest building material and are thought to protect bee colonies and/or enhance their growth [26]–[28]. Moreover, lactic acid bacteria (LAB), primarily belonging to the genera *Lactobacillus* and *Bifidobacterium*, have been described for several bee species, including honeybees (*Apis mellifera*) [29]–[32], bumblebees [32],[33], stingless bees [29] and several solitary bee species (e.g., *Xylocopa*, halictids, [19],[34]). LAB have been suggested to contribute to pollen fermentation within nests [30] and are known to also protect honeybees and bumblebees against pathogens [29],[35].

The microbiota of all social and solitary bees analyzed so far includes widespread LAB that are not host-specific. These LAB are closely related to flower-inhabiting, fructophilic lactobacilli or other lactobacilli found in the environment, which are likely obtained by bees when foraging at flowering plants [34]. In contrast, highly host-specific and diverged strains (i.e. strains of the Firm3, Firm4 and, to a lesser extent, Firm5 cluster) were reported for social bees, but not any other social insects [19],[32]. Based on this finding, McFrederick et al. [32] suggested that host-specificity of LAB is rare in Hymenoptera and may be maintained in social bees by spreading the symbionts among nestmates and transmitting them from one generation to the next via workers during colony fissioning.

While the microbial community of honeybees has received considerable attention, much less is known about the microbiota associated with stingless bees (Apidae: Meliponini) (but see [29]). Stingless bees represent the sister group of honeybees within the Apidae family, but in contrast to honeybees, they are restricted to tropical and subtropical regions, where they have achieved an impressive diversity with approximately 500 species described to date [36]. In a comparative study of the microbiota associated with several honeybee and stingless bee species, Vasquez et al. [29] found Firm4 and Firm5-associated LAB in South American, African and South East Asian stingless bee species, whereas LAB associated with Australian stingless bee species have not yet been investigated.

Here, we characterized the microbial community of three sympatric Australian stingless bee species (*Austroplebeia australis*, *Tetragonula carbonaria*, and *Tetragonula hockingsii*), with particular focus on the bee-associated LAB. According to the hypothesis that host-specific LAB are maintained by obligate colony fissioning (see McFrederick et al. (2013), we assume that LAB associated with the Firm3-5 clusters occur across all Meliponini. While the home ranges of the three species overlap broadly [37],[38], and they are similar in size and color (worker body size: [37],[38]), the two genera fall within different phylogenetic clades that diverged approximately 60 Mya ago [39]. The genus *Austroplebeia* (comprising five species) is endemic to Australia and Papua New Guinea and genetically more closely related to stingless bee lineages from the ancient African clade, whereas the closest relatives of the genus *Tetragonula* (comprising seven species in Australia) are found across Southeast Asia [39]. They also largely differ in their cuticular surface profiles [40] as well as their resource intake and nesting behavior, with only *Tetragonula* collecting [41] and incorporating substantial amounts of plant resins in their nest structures ([42]; Leonhardt SD, Drescher N, Wallace H, unpublished data). These differences in chemical ecology may result in a different nest and body environment for associated microbes.

## Methods

### Ethics statement

The stingless bee species investigated in this study are commonly found and not protected in Australia. As all bees were collected on private property, collecting permits were not required.

### Sampling of bees

Bee specimens for genetic analyses were collected from colonies located at the Glenmount Research Station in Buderim (South East Queensland, Australia) in March 2011 and 2012. All colonies had access to the same resource environment and faced the same ecological conditions, with a mixed rainforest and eucalypt forest as well as gardens included in their foraging range (approximately 500 m radius of the hive).

Specimens were collected from five colonies of *Tetragonula carbonaria*, four colonies of *Austroplebeia australis* and one colony of *Tetragonula hockingsii*, a species closely related to *T. carbonaria* [43], by placing a clean clear plastic bag over the hive entrance, thereby catching foragers leaving the nest. To kill the bees, the plastic bag was placed in a freezer for approximately 10 minutes. Following a close inspection of their bodies to exclude contamination with plant or hive material (e.g., pollen or resin), bees were then stored in 70% ethanol for molecular analysis.

### Bacterial tag-encoded FLX amplicon pyrosequencing (bTEFAP) and data analysis

DNA was extracted from six individual worker bees of all ten colonies sampled, respectively, using the MasterPure DNA Purification Kit (Epicentre Technologies) according to the manufacturer's instructions. For each colony, a pooled DNA sample was sent to an external service provider (Molecular Research LP, MR DNA, Shallowater, TX, USA) for bTEFAP with 16S rRNA primers Gray28F (5′-GAGTTTGATCNTGGCTCA-3′) and Gray519R (5′-GTNTTACNGCGGCKGCTG -3′) [44],[45]. A sequencing library was generated through one-step PCR with 30 cycles, using a mixture of HotStar and HotStar HiFidelity *Taq* polymerases (Qiagen). Sequencing extended from Gray28F, using a Roche 454 FLX instrument with Titanium reagents and procedures described at Molecular Research LP (http://www.mrdnalab.com/). Quality control and analysis of 454 reads was done in QIIME [46]. Low-quality ends of the sequences were trimmed with a sliding window size of 50 and an average quality cut-off of 25. Subsequently, all low quality reads (quality cut-off = 25) and sequences <200 bp were removed. High-quality reads were clustered into operational taxonomic units (OTUs) using a multiple OTU picking strategy with cdhit [47] and uclust [48], with 97% similarity cut-offs, respectively. For each OTU, the longest sequence was chosen as representative sequence (Data S1). Within the set of representative sequences, chimeras were identified using UCHIME (uchime_denovo) [49] and removed from further analysis. RDP classifier [50] and BLASTn against the NCBI database were used for taxonomy assignment. An OTU table was generated describing the occurrence of bacterial phylotypes within the samples (Table S1). OTUs were combined on the order level to display relative abundances.

### Phylogenetic analysis

For phylogenetic analysis, all OTUs with *Lactobacillus sp*. as the first BLASTn hit were selected (20 OTUs). The representative sequences for these OTUs were trimmed to 350 bp in order to remove potential low-quality ends that were not detected by the preceding quality-trimming steps (see above). The trimmed reads were combined with the *Lactobacillus* sequences used in McFrederick et al. [32] as well as the Meliponini-associated lactobacilli reported by Vasquez et al. [29]. The resulting 656 sequences were aligned to the SILVA SSU database [51] using the SINA aligner [52] (Data S2). An approximately-maximum-likelihood tree was reconstructed with FastTree 2.1 using the GTR model [53]. Local support values were estimated with the Shimodaira-Hasegawa test based on 1,000 resamples without reoptimizing the branch lengths for the resampled alignments [53].

## Results

### Bacterial community composition

Using bacterial tag-encoded FLX amplicon pyrosequencing (bTEFAP), we characterized the microbial communities associated with worker bees from four colonies of *A. australis*, five colonies of *T. carbonaria*, and one colony of *T. hockingsii*. In total, 139,771 reads were obtained (mean ± standard error  = 13,977±2,184 per sample), 126,919 of which passed quality filtering and chimera screening (mean ± standard error  = 12,692±1,843 per sample). Rarefaction analyses indicate that the microbiota associated with the individual colonies was exhaustively sampled, with the possible exception of the *T. carbonaria* colony H89 (Fig. S1). Based on 97% similarity clustering with cdhit [47] and uclust [48], the sequences were grouped into 241 OTUs (Table S1 and Data S1).

**Figure 1:**
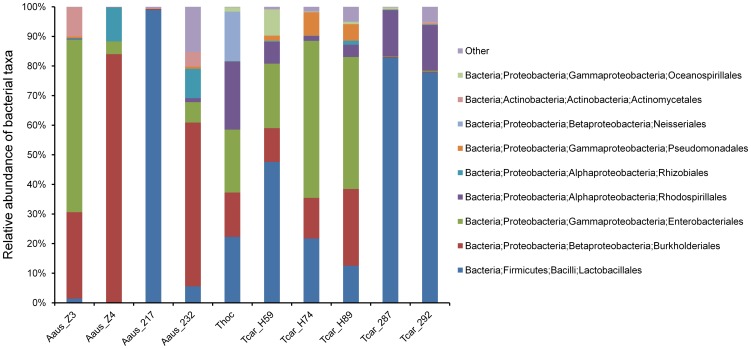
Bacterial community associated with three species of Australian stingless bees, as revealed by 16S tag-encoded FLX amplicon pyrosequencing. Different numbers denote different bee colonies. Aaus  =  *Austroplebeia australis*, Tcar  =  *Tetragonula carbonaria*, Thoc  =  *Tetragonula hockingsii*.

Lactobacillales (Firmicutes) as well as Beta-, Gamma-, and Alpha-Proteobacteria were the dominant taxa across colonies, but their relative abundance varied considerably within and between species (Fig. 1). One OTU associated with the genus *Ralstonia* (Beta-Proteobacteria, Burkholderiales; 99% similarity to *Ralstonia pickettii*) was consistently present across all colonies of the three bee species, two OTUs related to the genus *Pantoea* (Gamma-Proteobacteria, Enterobacteriales) were detected in six of the ten colonies across the three species, and one OTU associated with the family Acetobacteriaceae (Alpha-Proteobacteria, Rhodospirillales) occurred in all six *Tetragonula* colonies, but not in *A. australis* (Table S1). With the exception of the *A. australis* colony Z4, Lactobacillales were present in all colonies, with relative abundances ranging from 1.4 to 98.9% of the total microbiota.

### Phylogenetic affiliation of lactic acid bacteria (LAB)

**Figure 2:**
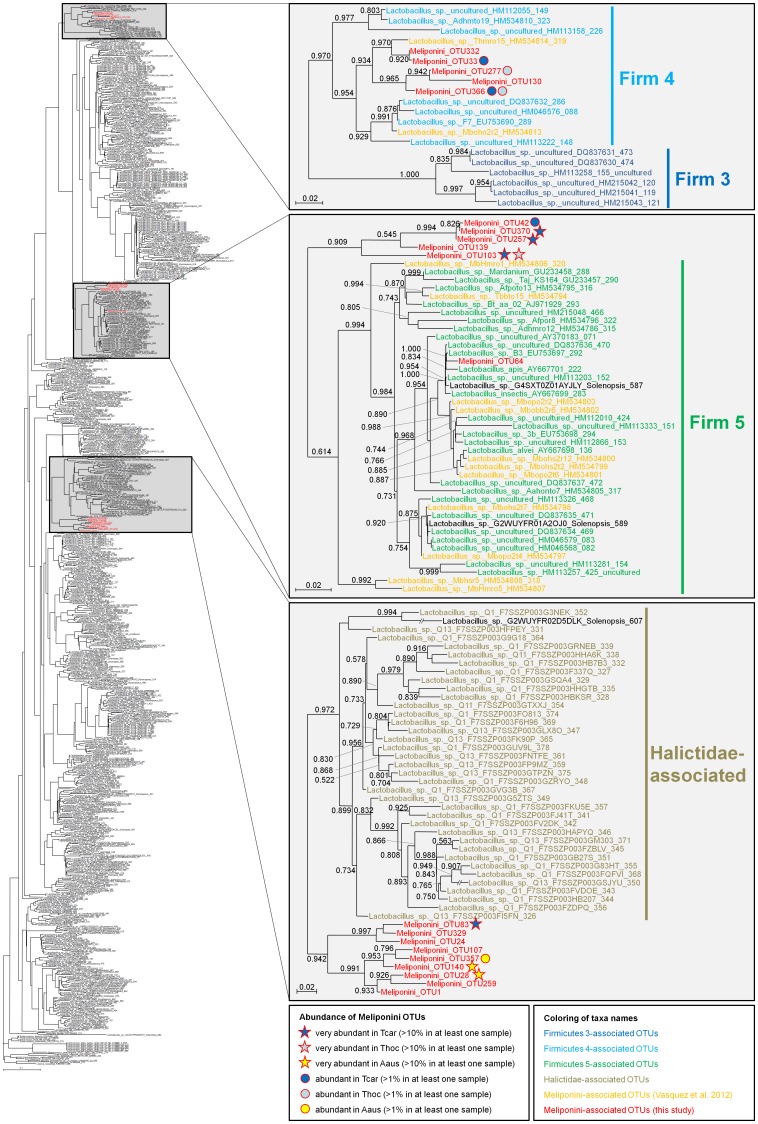
Phylogenetic affiliation of lactic acid bacteria associated with Australian stingless bees. Numbers at the tree nodes represent local support values based on the approximately maximum likelihood analysis performed in FastTree 2.1. Sequences obtained in the present study are highlighted in red font, stingless bee-associated sequences reported by Vasquez et al. (2012) are given in yellow font. Abundance of OTUs in the three investigated Australian stingless bee species is indicated by circles and asterisks, respectively, behind the OTU names. Aaus  =  *Austroplebeia australis*, Tcar  =  *Tetragonula carbonaria*, Thoc  =  *Tetragonula hockingsii*.

Among the 241 Meliponini-associated OTUs reported in this study, 20 were identified by RDP classification and BLASTn searches as members of the genus *Lactobacillus*. Phylogenetic analyses including 620 additional lactobacilli and outgroup sequences revealed the placement of the OTUs in four clusters (Fig. 2). (i) Five OTUs exclusively found in the two *Tetragonula* species were most closely related to a sequence obtained previously from a South East Asian stingless bee, *Trigona* sp. [29], and grouped within the bee-associated Firm4 cluster. (ii) A single sequence from the *T. carbonaria* colony 292 (OTU64) fell within the Firm5 cluster comprising honeybee- and stingless bee-associated LAB. (iii) Five OTUs only found in the two *Tetragonula* species formed the sister clade to the Firm5 cluster. And (iv) a monophyletic group of nine OTUs that were present in all three stingless bee species investigated in this study formed the sister clade to a large cluster of Halictidae-associated LAB.

## Discussion

While the microbial community of honeybees has been thoroughly investigated, the microbiota associated with stingless bees (Apidae: Meliponini) has only been addressed in a single comparative study with honeybees conducted by Vasquez et al. [29], which excluded Australian stingless bee taxa. Here we analyzed the microbiota associated with three Australian stingless bee species from two distinct phylogenetic lineages, in order to investigate the occurrence of host-specific LAB across stingless bees.

We found Lactobacillales (Firmicutes) as well as Beta-, Gamma-, and Alpha-Proteobacteria as the dominant bacterial taxa in all three stingless bee species. Samples from all ten colonies contained bacteria related to the genus *Ralstonia* (Burkholderiales), which are known as common pathogens [54],[55], but also as laboratory contaminants. Six of the ten colonies (including all species) additionally harbored bacteria associated with the genus *Pantoea* (Enterobacteriales, Gamma-Proteobacteria), which is a genus commonly found on plant roots, leaves [56] and flowers [57]. It has also been found in the hive environment and intestines of honeybees [58] [59]. Likewise, the family Acetobacteriaceae (Alpha-Proteobacteria, Rhodospirillales) that occurred in all six *Tetragonula* colonies (but not in *A. australis*) is often found in floral nectar and in the environment of bees (reviewed by [60]). Acetobacteriaceae belong to the acetic acid bacteria (AABs) and are known to break down carbohydrates in an acidic environment. They have recently been found to regulate the immune system homeostasis of *Drosophila* [61] and were suggested to be secondary symbionts across many insects [60]. They were also detected in the gut of a solitary bee species, *Osmia bicornis* [62]. In agreement with Koch et al. [20], we did not find the honeybee symbionts *Gilliamella apicola* and *Snodgrassella alvi*. The latter was implicated in the protection of bumblebees against *Crithidia bombi* [25].

Lactic acid bacteria (LAB) were present in nine out of ten colonies, but only the two *Tetragonula* species contained LAB that are closely related to other stingless bees and honeybee associated LAB, i.e. belong to the host-specific Firm4 and Firm5 clusters. Interestingly, neither our study nor the study of Vasquez et al. [29] found LAB of the Firm3 cluster in stingless bees. The Firm3 cluster is the most derived cluster of LAB associated with bees and may be honey- and bumblebee-specific, while Firm4–5 LAB are shared by honeybees, bumblebees and stingless bees across geographical regions. However, the absence of LAB of the Firm4–5 cluster in *A. australis* indicates that they are not present in all corbiculate bee species that propagate through colony fissioning as suggested by McFederick et al. [32].

In addition to the Firm4–5 clusters, we identified a novel cluster of LAB that is closely related to Halictidae-associated LAB in both *A. australis* and the two *Tetragonula* species. Despite its phylogenetic affiliation with the *Lactobacillus buchneri* group, this monophyletic group (comprising LAB associated with Halictidae and Meliponini) may represent a novel host-specific clade of bee-associated LAB.

Considering the occurrence of host-specific LAB (particularly Firm4–5) across corbiculate bees, their presence in stingless bees appears to be the ancestral state, with *A. australis* having secondarily lost the Firm4–5 cluster. This agrees with earlier studies detecting LAB of the Firm4–5 cluster in other stingless bees of the genera *Trigona, Melipona*, and *Meliponula* [29]. Given the close phylogenetic affiliation of *Austroplebeia* with the African genus *Lisotrigona* [39], it will be interesting to characterize the microbial community of additional species in these two genera, in order to find out how widespread the loss of Firm4–5 LAB is across stingless bees. Furthermore, investigating the distribution of LAB of the Halictidae-Meliponini cluster identified in this study across social and non-social bees may yield novel insights into the occurrence of host-specific LAB in bees. The widespread occurrence and potential host specificity of LAB in stingless bees suggests an important function in the protection against pathogens [29],[35] or in pollen fermentation within nests [30], as has been demonstrated for honeybees.

Our analysis of the microbiota associated with three Australian stingless bee species shows that the LAB community associated with stingless bees resembles that associated with honeybees, but lacks LAB of the highly host-specific Firm3 cluster and instead comprises an additional clade of likely host-specific LAB that form a sister clade to a large cluster of Halictidae-associated lactobacilli. This finding suggests that LAB are of similar ecological importance to stingless bees as they are to other corbiculate bees, but that their composition depends on the phylogenetic background and geographic region of their hosts. Therefore, stingless bees represent interesting organisms for understanding the evolutionary history of the bee-LAB association.

## Supporting Information

Figure S1 **Rarefaction analysis with the sequencing data for 10 colonies belonging to three different species of Australian stingless bees.** Different numbers denote different bee colonies. Aaus  =  *Austroplebeia australis*, Tcar  =  *Tetragonula carbonaria*, Thoc  =  *Tetragonula hockingsii*.
(TIF)

Table S1 **Abundance of 241 operational taxonomic units (OTUs) across ten colonies of three different Australian stingless bee species (Aaus  =** ***Austroplebeia australis*** **, Tcar  =** ***Tetragonula carbonaria*** **, Thoc  =** ***Tetragonula hockingsii*** **).** Taxonomic assignment was done with the RDP classifier based on the representative sequences for the OTUs. OTUs associated with the genus *Lactobacillus* (BLASTn results) are highlighted in bold print.
(XLSX)

Data S1 **Representative sequences for 241 OTUs identified across ten colonies of three different Australian stingless bee species.** Each sequence is identified by the OTU number, the species and colony it was found in (Aaus  =  *Austroplebeia australis*, Tcar  =  *Tetragonula carbonaria*, Thoc  =  *Tetragonula hockingsii*), and a unique sequence identifier. If an OTU was detected in multiple colonies, only one colony/species is indicated.
(FASTA)

Data S2 **Alignment of 656 lactobacilli and outgroup sequences used for the phylogenetic analyses of Meliponini-associated lactic acid bacteria.** The sequences obtained in this study were combined with the *Lactobacillus* sequences used in McFrederick et al. [32] as well as the Meliponini-associated lactobacilli reported by Vasquez et al. [29], and the resulting sequence set was aligned to the SILVA SSU database [51] using the SINA aligner [52].
(FASTA)
