# Supplementary figures and images for: Microbial Communities of Three Sympatric Australian Stingless Bee Species
Source: PLoS One. 2014 Aug 22;9(8):e105718. doi: 10.1371/journal.pone.0105718 (PMC4141829; doi:10.1371/journal.pone.0105718)

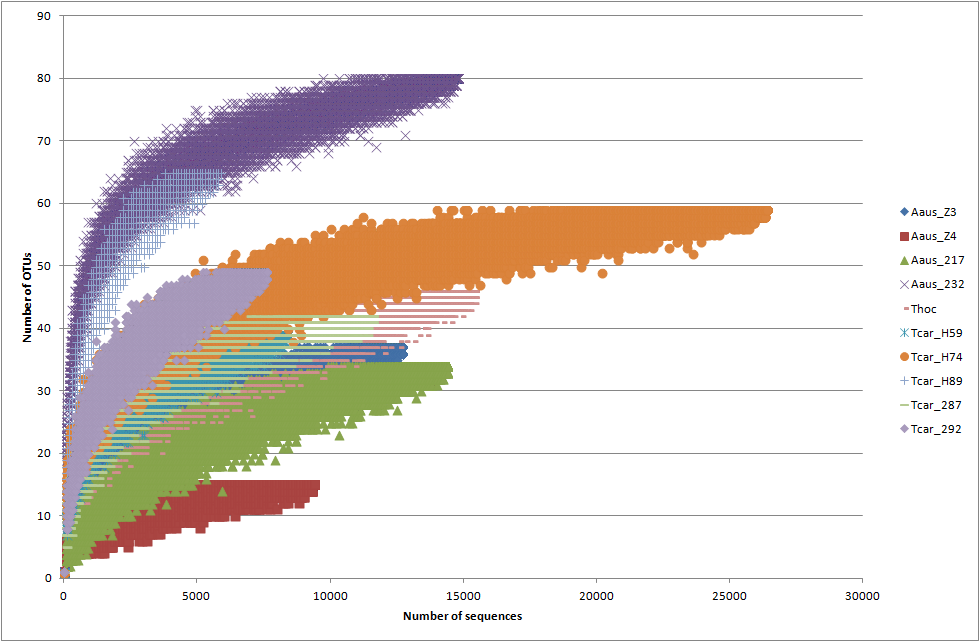

Supplement: Figure S1 — Rarefaction analysis with the sequencing data for 10 colonies belonging to three different species of Australian stingless bees. Different numbers denote different bee colonies. Aaus = Austroplebeia australis, Tcar = Tetragonula carbonaria, Thoc = Tetragonula hockingsii. (TIF) [file pone.0105718.s001.tif]
